# Agreement between molecular subtyping and surrogate subtype classification: a contemporary population-based study of ER-positive/HER2-negative primary breast cancer

**DOI:** 10.1007/s10549-019-05378-7

**Published:** 2019-08-20

**Authors:** Christine Lundgren, Pär-Ola Bendahl, Åke Borg, Anna Ehinger, Cecilia Hegardt, Christer Larsson, Niklas Loman, Martin Malmberg, Helena Olofsson, Lao H. Saal, Tobias Sjöblom, Henrik Lindman, Marie Klintman, Jari Häkkinen, Johan Vallon-Christersson, Mårten Fernö, Lisa Rydén, Maria Ekholm

**Affiliations:** 1 Department of Oncology, Jönköping, Region Jönköping County, and Department of Clinical and Experimental Medicine, Linköping University, Linköping, Sweden; 2 Department of Clinical Sciences Lund, Division of Oncology and Pathology, Lund University, Lund, Sweden; 3 Department of Laboratory Medicine Lund, Division of Translational Cancer Research, Lund University, Lund, Sweden; 4 Department of Hematology, Oncology and Radiation Physics, Skåne University Hospital, Lund, Sweden; 5 Department of Immunology, Genetics and Pathology, Uppsala University, Uppsala, Sweden; 6 Department of Clinical Sciences Lund, Division of Surgery, Lund University, Lund, Sweden

**Keywords:** Breast cancer, Intrinsic subtype, Molecular subtyping, Surrogate marker, Gene expression

## Abstract

**Purpose:**

Oestrogen receptor-positive (ER+) and human epidermal receptor 2-negative (HER2–) breast cancers are classified as Luminal A or B based on gene expression, but immunohistochemical markers are used for surrogate subtyping. The aims of this study were to examine the agreement between molecular subtyping (MS) and surrogate subtyping and to identify subgroups consisting mainly of Luminal A or B tumours.

**Methods:**

The cohort consisted of 2063 patients diagnosed between 2013–2017, with primary ER+/HER2– breast cancer, analysed by RNA sequencing. Surrogate subtyping was performed according to three algorithms (St. Gallen 2013, Maisonneuve and our proposed Grade-based classification). Agreement (%) and kappa statistics (κ) were used as concordance measures and ROC analysis for luminal distinction. Ki67, progesterone receptor (PR) and histological grade (HG) were further investigated as surrogate markers.

**Results:**

The agreement rates between the MS and St. Gallen 2013, Maisonneuve and Grade-based classifications were 62% (κ = 0.30), 66% (κ = 0.35) and 70% (κ = 0.41), respectively. PR did not contribute to distinguishing Luminal A from B tumours (auROC = 0.56). By classifying HG1-2 tumours as Luminal A-like and HG3 as Luminal B-like, agreement with MS was 80% (κ = 0.46). Moreover, by combining HG and Ki67 status, a large subgroup of patients (51% of the cohort) having > 90% Luminal A tumours could be identified.

**Conclusions:**

Agreement between MS and surrogate classifications was generally poor. However, a post hoc analysis showed that a combination of HG and Ki67 could identify patients very likely to have Luminal A tumours according to MS.

## Background

Almost 20 years have passed since the breast cancer molecular intrinsic subtypes, based on patterns of gene expression, were first presented [1, 2]. The majority of oestrogen receptor (ER)-positive and human epidermal receptor 2 (HER2)-negative (ER+/HER2–) breast cancers are classified as Luminal A or Luminal B. An important difference between these groups is that patients with Luminal B tumours have a higher risk of relapse [2] and are therefore often recommended adjuvant chemotherapy in addition to endocrine therapy [3]. During the last decade, several commercial multigene assays have been introduced as tools for estimating the risk of recurrence (ROR) and for selecting patients for whom adjuvant chemotherapy can be omitted [4–8]. One of these tests, PAM50/Prosigna^®^, provides information on both intrinsic subtype and a ROR score, based on gene expression and tumour size [4, 9].

Surrogate subgroups based on hormone receptor expression, proliferation and HER2 status were introduced a decade ago as a tool for risk stratification and guidance of adjuvant therapy [10]. In the St. Gallen 2013 surrogate subtype classification, a combination of the routine pathological markers ER, PR and HER2 and the proliferation marker Ki67 is used to classify tumours into the intrinsic subtypes. Ki67 (high/low) and PR (high/low) are used to separate Luminal A-like from Luminal B-like tumours [11]. The following year, Maisonneuve and colleagues proposed a new surrogate definition for the luminal intrinsic subtypes by introducing an intermediate Ki67 group [12]. They also showed that PR was a discriminator for prognosis only in tumours with intermediate Ki67. In both St. Gallen 2013 and Maisonneuve classifications, the cut-off for high PR was set at ≥ 20%. The classification proposed by Maisonneuve showed improved stratification compared with St. Gallen 2013 in terms of long-term outcome (distant disease-free survival). Furthermore, in their large cohort of almost 10 000 breast cancer patients, they found that patients with poorly differentiated [histological grade (HG) 3] Luminal A-like tumours had a prognosis similar to that of patients with Luminal B-like tumours, whereas patients with well-differentiated (HG1) Luminal B-like tumours had a prognosis similar to that of Luminal A-like tumours. Similar results were obtained in a study (*n* = 671) by our research group, where HG was shown to add prognostic information in terms of distant disease-free survival to the St. Gallen 2013 classification [13]. In the St. Gallen consensus statement 2017, the surrogate classification was not described in detail; Luminal A-like tumours are defined as having high ER/PR, *clearly low* Ki67, HG1, whereas Luminal B-like tumours have lower ER/PR, *clearly high* Ki67, HG3. There is no clear definition for the intermediate group and the use of molecular assays for these tumours has been suggested as a tool for improved risk stratification [3].

By using molecular assays, more patients can be spared adjuvant chemotherapy, but since these tests are associated with significant costs, the routinely used pathological morphology and immunohistochemical (IHC) markers still form the basis for the adjuvant treatment decision for most patients. In an ongoing Swedish population-based observational study, SCAN-B (Sweden Cancerome Analysis Network—Breast) [14, 15], primary breast cancer tissue samples are classified into molecular intrinsic subtypes by RNA sequencing, using the PAM50 genes and an implementation of the nearest-centroid method.

We hypothesised that the concordance of the molecular subtypes (Luminal A and Luminal B) to the clinicopathological surrogate subtypes (Luminal A-like and Luminal B-like) could be improved by prioritising HG when subtyping tumours and only including the other markers in HG2 tumours. In this Grade-based classification, HG1 tumours were therefore classified as Luminal A-like and HG3 tumours as Luminal B-like. Ki67 and PR were then evaluated as discriminators in HG2 tumours. For definition of the different surrogate classifications, see Appendix Table 4.

The primary aim of this study was to compare the concordance between MS classification (as assessed by RNA sequencing within the SCAN-B project) and different surrogate classifications, namely St. Gallen 2013, Maisonneuve and our proposed Grade-based classification, for ER+/HER2– tumours. Secondary aims were to investigate the discriminatory value of each of the markers Ki67, PR and HG, and moreover to conduct an exploratory analysis to define subgroups consisting mainly of Luminal A or Luminal B tumours.

## Materials and methods

### Patients

The study cohort consists of patients consecutively included in SCAN-B (Sweden Cancerome Analysis Network—Breast, ClinicalTrials.gov ID: NCT02306096), a multicentre study that was initiated in 2010 with the long-term aim of prospectively analysing breast tumour tissue by genetic methods for translational research and for the development and implementation of molecular assays. There were eight participating hospitals during the years 2013–2015 and nine from 2015 and onwards. These hospitals cover approximately 25% of all patients diagnosed with breast cancer in Sweden [16]. The genomic analyses were all centrally assessed at one institution (Lund, Sweden). All patients who are considered to be able to provide an informed consent are asked to participate and > 90% of invited patients accept to be included in the trial, hence the SCAN-B cohort is considered to be population-based. For about 75% of the participating patients, tumour tissue is available for further gene expression and biomarker analyses [14, 15]. Fresh breast cancer tumour tissue is collected during the routine pathology preparation and analysed by RNA sequencing. Clinicopathological variables and patient-related characteristics for all patients diagnosed in Sweden are recorded in the Swedish National Quality Register for Breast Cancer (NKBC), which has a coverage rate of almost 100% [16]. Data from this register are made available for enrolled patients and transferred to SCAN-B.

**Fig. 1:**
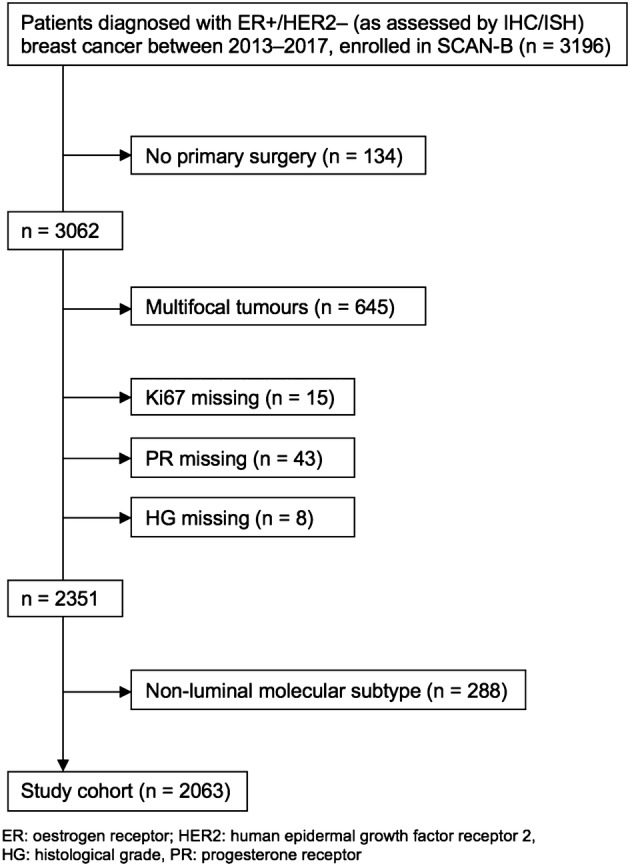
Flow chart of the study cohort

All patients with ER+/HER2– breast cancers enrolled in SCAN-B during 2013–2017 (*n* = 3196) were identified. Patients diagnosed before 2013 were not included as register data on Ki67 for these are scarce. Patients who did not undergo primary surgery (*n* = 134) were excluded. The rationale for this was that Ki67 assessed on core biopsies is not comparable to Ki67 assessed on surgical specimens [17, 18]. Moreover, histological grade should not be assessed on core biopsies according to Swedish guidelines. The register data for multifocal tumours are known to be associated with uncertainties, so these were excluded (*n* = 645), as were tumours with missing data for any of Ki67, PR and HG (*n* = 66). Because we aimed to investigate agreement only for luminal tumours, tumours with a non-luminal molecular subtype were excluded (*n* = 288), resulting in a study cohort of 2063 patients (Fig. 1). Tumour and patient characteristics are summarised in Table 1.

**Table 1:** Tumour and patient characteristics of the included patients diagnosed with ER+/HER2– tumours by IHC/ISH with a molecular luminal profile (*n* = 2063)

Characteristics	Number of patients *n* (%)
Tumour size (mm)	
≤ 20	1451 (71)
> 20 but ≤ 50	557 (27)
> 50	46 (2)
Missing	9
Number of positive nodes	
0	1440 (71)
1–3	509 (25)
4–9	66 (3)
≥ 10	29 (1)
Missing	19
PR^a^	
Positive	1780 (86)
Negative	283 (14)
Histological grade	
1	458 (22)
2	1202 (58)
3	403 (20)
Ki67	
Low (< 14%)	523 (25)
Intermediate (14–19%)	443 (22)
High (≥ 20%)	1097 (53)
Age	
< 40	14 (1)
≥ 40 but < 50	106 (5)
≥ 50 but < 60	356 (17)
≥ 60	1587 (77)
Histopathological tumour type	
Ductal/no special type	1619 (79)
Lobular	295 (14)
Other	148 (7)
Missing	1

^a^*PR* progesterone receptor. Regarded as positive if defined as positive in the Swedish National Quality Register for Breast Cancer, or a value of PR > 10%

### Tumour processing and subtype classification

Fresh tumour tissue was collected for SCAN-B at local pathology departments in conjunction with the regular clinical routines of preparing formalin-fixed specimens for routine histopathology analyses. Fresh tissue was preserved in RNAlater and continuously sent for further processing at the SCAN-B laboratory (Lund, Sweden) where RNA/DNA was extracted. In general, RNA was sequenced within a week after surgery. The intrinsic subtype was determined by a nearest-centroid implementation using the PAM50 genes and centroids as described by Parker et al. [4]. To avoid cohort dependence when assigning PAM50 subtype, fixed reference cohorts for gene centring were selected to match the original training population used by Parker et al. Tumour specimens were then assigned to subtype, namely Luminal A, Luminal B, HER2-enriched, Basal-like or Normal-like, according to the most frequent nearest centroid. Tumours were denoted as Unclassified when the correlation coefficients were below 0.2 to all of the subtype centroids. Methods of tissue preparation and analyses have been described elsewhere [14, 19].

In accordance with Swedish guidelines, ER/PR-positivity was defined as > 10% of positively stained tumour cells of the tissue section assessed by IHC. Ki67 was assessed by counting the percentage of positively stained nuclei in at least 200 cells in hotspots. HER2 was assessed by IHC and scored as 0, 1+ , 2+ or 3+ and 2+ cases were further analysed by in situ hybridisation (ISH). HER2+ was defined as score 3+ alone or score 2+ in combination with her2 gene amplification by ISH [20]. Histological grade was determined in accordance with the work of Elston-Ellis [21]. The tumours were classified according to the surrogate definitions of St. Gallen 2013, Maisonneuve and our proposed Grade-based classification (Appendix Table 4).

### Statistical analyses and ethics

Percentage agreement and kappa statistics were used for concordance analyses. Kappa (κ) is a measure of concordance between categorical variables, which adjusts for the amount of agreement that would be expected by chance. The most commonly used interpretation of κ is as follows: ≤ 0.40 poor/fair agreement; 0.41–0.60 moderate agreement; 0.61–0.80 substantial agreement and > 0.80 almost perfect agreement  [22]. To evaluate the capacity to distinguish between Luminal A and Luminal B, ROC analysis was performed, and the area under the ROC curve (auROC) was calculated. For kappa and auROC, 95% confidence intervals (CIs) were reported. McNemar’s test was performed to evaluate the difference among the luminal distributions between MS and surrogate classification (*p* value < 0.05 considered statistically significant). Statistical analyses were performed using IBM SPSS Statistics for Macintosh, Version 25.0.

## Results

In the present cohort, 39% (*n* = 808) and 61% (*n* = 1255) of the tumours were classified as Luminal A-like and Luminal B-like, respectively, according to St. Gallen 2013. The corresponding figures were 43% (*n* = 894) and 57% (*n* = 1169) for the Maisonneuve classification, and 49% (*n* = 1004) and 51% (*n* = 1059) for the Grade-based classification. By MS, 71% (*n* = 1458) of the tumours were assessed as Luminal A and 29% (*n* = 605) as Luminal B. There were significant differences of the luminal distributions of MS compared with the St. Gallen 2013, Maisonneuve and Grade-based classifications, respectively (all *p* values < 0.001).

### Agreement between MS and the three surrogate classifications

The agreements between MS and the different surrogate classifications were as follows: St. Gallen 2013: 62% [κ = 0.30, (95% CI 0.27–0.34)], Maisonneuve: 66% [κ = 0.35, (95% CI 0.32–0.38)] and Grade-based: 70% [κ = 0.41, (95% CI 0.37–0.44)].

### Ki67, PR and histological grade (HG) as surrogate markers

The rates of Luminal A (MS) in tumours with low (< 14%), intermediate (14–19%) and high (≥ 20%) Ki67 were 96%, 86% and 53%, respectively (Table 2). The proportions of low, intermediate and high Ki67 in this cohort were 25%, 22% and 53%, which differ from the distribution in the work of Maisonneuve et al. (34%, 24% and 42%) [12]. We therefore conducted an exploratory analysis using the same Ki67 percentiles, resulting in a slight change of the cut-offs: low: < 16%, intermediate: 16–23% and high: ≥ 24%. After this adjustment, the agreement between the Maisonneuve surrogate classification based on the new Ki67 cut-offs and MS increased to 73% [κ = 0.44, (95% CI 0.40–0.48)]. The corresponding figure for the Grade-based classification was 75% [κ = 0.46, (95% CI 0.43–0.50)].

**Table 2:** Ki67 subgroup distribution and proportion of molecular luminal subtypes for all tumours and for tumours with HG1, HG2 and HG3

Molecular subtype	Ki67 < 14%, *n* (%)	Ki67 14–19%, *n* (%)	Ki67 ≥ 20%, *n* (%)	Total, *n* (%)
All tumours (*n* = 2063)				
Luminal A	500 (96)	379 (86)	579 (53)	1458 (71)
Luminal B	23 (4)	64 (14)	518 (47)	605 (29)
Total	523 (25)	443 (22)	1097 (53)	2063 (100)
HG1 tumours (*n* = 458)				
Luminal A	211 (97)	116 (89)	96 (87)	423 (92)
Luminal B	7 (3)	14 (11)	14 (13)	35 (8)
Total	218 (48)	130 (28)	110 (24)	458 (100)
HG2 tumours (*n* = 1202)				
Luminal A	282 (95)	255 (86)	389 (64)	926 (77)
Luminal B	15 (5)	43 (14)	218 (36)	276 (23)
Total	297 (25)	298 (25)	607 (51)	1202 (100)
HG3 tumours (*n* = 403)				
Luminal A	7 (88)	8 (53)	94 (25)	109 (27)
Luminal B	1 (13)	7 (47)	286 (75)	294 (73)
Total	8 (2)	15 (4)	380 (94)	403 (100)

In total, 16% (*n* = 330) of the tumours were PR-low (cut-off < 20%) and 59% (*n* = 194) of these were Luminal A by MS, compared with 73% (*n* = 1264) in PR-high (cut-off ≥ 20%) tumours (*n* = 1733). To evaluate the capacity of PR to distinguish between Luminal A and B tumours, ROC analyses were performed. In the surrogate algorithms presented in this paper, PR is used as a luminal discriminator in the following groups: tumours with low Ki67 (St. Gallen 2013, *n* = 523), intermediate Ki67 (Maisonneuve, *n* = 443) and intermediate Ki67 and HG2 (Grade-based, *n* = 298). The auROC values for PR (percentages from 0 to 100%) for these three groups were 0.51 (95% CI 0.39–0.63), 0.56 (95% CI 0.48–0.63) and 0.56 (95% CI 0.46–0.65), respectively.

The proportions of HG1, HG2 and HG3 in the overall cohort were 22%, 58% and 20%, respectively, and the distribution of Luminal A (MS) in the different HG categories was as follows: HG1: 92%, HG2: 77% and HG3: 27% (Table 2). In an exploratory analysis, we classified HG1-2 tumours as Luminal A-like and HG3 tumours as Luminal B-like, and the agreement with the corresponding MS increased to 80% [κ = 0.46 (95% CI 0.41–0.50)]. When including only HG1 and HG3 tumours, the agreement was 83% [κ = 0.66 (95% CI 0.61–0.71)] (*n* = 861).

### Exploratory analysis of combining HG and Ki67 as a surrogate classification

To identify subgroups in which MS may be omitted due to a high proportion of molecular subtypes of either Luminal A or B, tumours were divided into nine subgroups based on HG (1–3) and Ki67 [low (< 14%), intermediate (14–19%), high (≥ 20%)]. PR was not included in this refined algorithm due to its low capacity to distinguish between the luminal tumours presented above. Six of the subgroups (HG1 with any Ki67 category, HG2 with low and intermediate Ki67 and HG3 with low Ki67), comprising 51% (*n* = 1061) of the tumours, were found to consist of 91% Luminal A tumours, as assessed by MS. The distribution of Luminal A in the nine subgroups defined by Ki67 and HG is presented in Table 3.

**Table 3:** Proportion of Luminal A tumours, according to molecular subtyping, in subgroups generated by combining histological grade (HG1–3) and Ki67 in three categories (according to Maisonneuve et al. [12])

Ki67	HG1	HG2	HG3
Low (< 14%)	97% Luminal A (*n* = 211 of 218)	95% Luminal A (*n* = 282 of 297)	88% Luminal A (*n* = 7 of 8)
Intermediate (14–19%)	89% Luminal A (*n* = 116 of 130)	86% Luminal A (*n* = 255 of 298)	53% Luminal A (*n* = 8 of 15)
High (≥ 20%)	87% Luminal A (*n* = 96 of 110)	64% Luminal A (*n* = 389 of 607)	25% Luminal A (*n* = 94 of 380)
Total	92% Luminal A (*n* = 423 of 458)	77% Luminal A (*n* = 926 of 1202)	27% Luminal A (*n* = 109 of 403)

## Discussion

In this population-based contemporary study of breast cancer, including more than 2000 prospectively assessed breast tumours, we show that there was poor agreement between different surrogate definitions of luminal tumour subtypes and molecular PAM50 (Parker algorithm) [4] subtyping. Considerably more tumours were Luminal A as determined by MS than by the surrogate classifications. However, one should not expect to find perfect surrogates for the molecular subtypes since the surrogate algorithms are based on IHC assessment of protein levels, whereas the MS is based on the measurement of mRNA transcript levels of corresponding genes (*ESR1*, *PGR* and *MKI67*) as well as of additional genes within the PAM50 panel, depicting the underlying biological signalling pathways. By simply designating HG1-2 as Luminal A-like and HG3 as Luminal B-like, the agreement with MS was superior to all of the surrogate classifications presented in this paper. The discriminatory importance of HG is not unexpected, being reported both in a previous study from our research group and by Maisonneuve et al. [12, 13].

The reproducibility of HG has, however, been shown to be only moderate [23]. Regarding Ki67, the issues of inter-laboratory variability and cut-off levels are also well known [24, 25]. In an exploratory analysis, we used the same percentiles for low, intermediate and high Ki67 as in the study by Maisonneuve et al. [12], by which the agreement with MS improved (from 66 to 73%). This emphasises the importance of critical reflection over Ki67 cut-offs in the local laboratory. Moreover, only 47% of the tumours in the high-Ki67 category were shown to be Luminal B according to MS, which raises the question of whether a cut-off of 20% is too low to be able to identify Luminal B-like tumours and thereby identify patients with hormone receptor-positive tumours who might benefit from additional adjuvant chemotherapy. We found no added value of PR in the surrogate classifications. However, since there were no follow-up data available for this cohort, we were not able to evaluate PR as a prognostic marker.

When considering the lack of agreement in the results presented here, one should also be aware of the concerns regarding agreement between different multigene tests. Bartlett et al. compared different multiparameter tests regarding risk classification and intrinsic subtyping and found a rate of discordant results among Prosigna^®^, Blueprint^®^ and MammaTyper^®^ as high as 41% for tumour subtyping [26]. Similar discordant results from the application and comparison of different commercial gene signatures on RNA sequencing data were found in our own study of the SCAN-B cohort (Vallon-Christersson et al. submitted).

As current commercially available variants of molecular assays are associated with considerable cost, it would be desirable to identify patients who could be spared chemotherapy based on less costly molecular tests or from routine pathology only. We found that 91% of the tumours with HG1 (irrespective of Ki67) and HG2 tumours with Ki67 < 20% were identified to be Luminal A by MS. These comprised slightly more than half of the overall cohort; the cost-effective use of commercial molecular assays for further stratification, in the absence of negative prognostic features such as age, nodal involvement or large tumours, may therefore be limited in this group. Tumours with HG3 and low Ki67 were also found to be mostly Luminal A; however, owing to the small numbers in this subgroup (*n* = 8), this result is considered unreliable.

The strengths of this study are the large cohort of more than 2000 patients, the fact that patients were prospectively included and that the study cohort can be regarded as population-based. A limitation is that the MS definition in the SCAN-B project has not yet been clinically validated. However, as pointed out by others, a nearest-centroid classification that appropriately addresses cohort composition to match the original study population is expected to be almost equivalent to the commercial assay [27]. Moreover, because of the short follow-up, no evaluation of the prognostic value of the presented results was performed.

## Conclusion

The present study indicates poor agreement between surrogate classifications and MS of luminal breast cancer tumours. By combining HG and Ki67, a large subgroup of the patients could be identified as Luminal A assessed by MS. This group of patients may not benefit from the use of molecular assays, especially if other clinicopathological factors indicate a low risk of recurrence. We are aware of the issue regarding the poor reproducibility of Ki67 and HG assessments, favouring MS. Nonetheless, the results of this study offer new insights into how to use MS in combination with histopathological markers in a clinical context, but further studies including adequate follow-up data are needed to correlate these findings with patient outcome.

## Appendix

See Table 4.

**Table 4:** Definition of different surrogate subtyping classifications for ER+/HER2– breast cancer tumours

Clinicopathological surrogate definition	Characteristics
St. Gallen 2013 [11]	
Luminal A-like	Low Ki67 (< 20%) and high PR (≥ 20%)
Luminal B-like	High Ki67 (≥ 20%) and/or low PR (< 20%)
Maisonneuve [12]	
Luminal A-like	Low Ki67 (< 14%) *or* Intermediate Ki67 (14–19%) and high PR (≥ 20%)
Luminal B-like	High Ki67 (≥ 20%) *or* Intermediate Ki67 (14–19%) and low PR (< 20%)
St. Gallen 2017 [3]	
Luminal A-like	High ER/PR, clearly low Ki67, HG1
Intermediate	Uncertainties persist about risk and degree of responsiveness to endocrine and cytotoxic therapies
Luminal B-like	Lower ER/PR, clearly high Ki67, HG3
Grade-based classification	
Luminal A-like	HG1 *or* HG2 and low Ki67 (< 14%) *or* HG2 and intermediate Ki67 (14–19%) and high PR (≥ 20%)
Luminal B-like	HG3 *or* HG2 and high Ki67 (≥ 20%) *or* HG2 and intermediate Ki67 (14–19%) and low PR (< 20%)

*ER* oestrogen receptor, *HER2* human epidermal growth factor receptor 2, *HG* histological grade, *PR* progesterone receptor
